# Supplementary material for: Emission Characteristics of Polycyclic Aromatic Hydrocarbons and Nitro-Polycyclic Aromatic Hydrocarbons from Open Burning of Rice Straw in the North of Vietnam
Source: Int J Environ Res Public Health. 2019 Jul 2;16(13):2343. doi: 10.3390/ijerph16132343 (PMC6651601; doi:10.3390/ijerph16132343)
Supplement: Supplementary file 1 [file ijerph-16-02343-s001.pdf]

Table S1: Sampling information for $PM_{2.5}$

BG Samples	Sampling time (minutes)	Volume (m³)	Mass (mg)	Concentration (µg/m³)	BB Samples	Sampling time (minutes)	Volume (m³)	Mass (mg)	Concentration (µg/m³)
BG1	117	0.585	0.346	591.45	BB1	35	0.175	1.322	7554.29
BG2	120	0.6	0.326	543.33	BB2	31	0.155	2.464	15896.77
BG3	120	0.6	0.294	490.00	BB3	40	0.2	0.844	4220.00
BG4	120	0.6	0.32	533.33	BB4	21	0.105	0.972	9257.14
BG5	120	0.6	0.34	566.67	BB5	35	0.175	0.538	3074.29
BG6	120	0.6	0.51	850.00	BB6	20	0.1	1.842	18420.00
BG7	120	0.6	0.1	166.67	BB7	42	0.21	0.078	371.43
BG8	120	0.6	0.112	186.67	BB8	21	0.105	3.072	29257.14
BG9	120	0.6	0.068	113.33	BB9	26	0.130	0.612	4707.69
BG10	120	0.6	0.066	110.00	BB10	31	0.155	0.776	5006.45
BG11	120	0.6	0.096	160.00	BB11	25	0.125	1.058	8464.00
BG12	120	0.6	0.284	473.33	BB12	24	0.12	1.350	11250.00
BG13	120	0.6	0.251	418.33	BB13	33	0.165	2.010	12181.82
BG14	120	0.6	0.255	425.00	BB14	30	0.15	3.180	21200.00

Table S2: Sampling information for TSP

BG Samples	Sampling time (minutes)	Volume (m³)	Mass (mg)	Concentration (μg/m³)	BB Samples	Sampling time (minutes)	Volume (m³)	Mass (mg)	Concentration (μg/m³)
BG1	120	120	24.40	203.4	BB1	35	35	152.8	4365.3
BG2	120	120	33.34	277.8	BB2	31	31	270.3	8718.8
BG3	120	120	35.25	293.8	BB3	40	40	74.3	1857.3
BG4	120	120	41.23	343.6	BB4	21	21	132.7	6317.5
BG5	120	120	29.24	243.7	BB5	35	35	57.2	1634.5
BG6	120	120	28.98	241.5	BB6	20	20	313.6	15678.8
BG7	120	120	11.52	96.0	BB7	42	42	15.5	368.0
BG8	120	120	13.44	112.0	BB8	21	21	785.7	37413.0
BG9	120	120	9.12	76.0	BB9	26	26	60.5	2327.0
BG10	120	120	12.60	105.0	BB10	31	31	152.7	4926.0
BG11	120	120	16.95	141.3	BB11	25	25	507.5	20300.0
BG12	120	120	13.65	113.8	BB12	24	24	763.5	19576.9
BG13	120	120	10.20	85.0	BB13	33	33	364.8	11053.9
BG14	120	120	10.10	84.2	BB14	30	30	576.6	19218.7
